# Editorial: Pulmonary hypertension in atrial septal defect

**DOI:** 10.3389/fcvm.2025.1589305

**Published:** 2025-03-24

**Authors:** K. Krishnathasan, I. Rafiq, K. Dimopoulos

**Affiliations:** ^1^ Adult Congenital Heart Centre and Centre for Pulmonary Hypertension, Royal Brompton Hospital, Part of GSTT, London, United Kingdom; ^2^ National Heart and Lung Institute, Imperial College London, London, United Kingdom

**Keywords:** genetics, congenital heart defect (CHD), atrial septal defect, pulmonary arterial hypertension, pulmonary arterial hypertension associated with congenital heart disease

**Editorial on the Research Topic**
Pulmonary hypertension in atrial septal defect

This issue of Frontiers covers important topics in the area of pulmonary arterial hypertension associated with congenital heart disease (PAH-CHD). This is a complex heterogeneous population that poses major challenges in terms of diagnosis and treatment. PAH-CHD is categorised into four major clinical/pathophysiological groups: Group A (Eisenmenger syndrome, the extreme end of the spectrum with severe PAH and cyanosis), Group B (PAH with a “predominant” left-to-right shunt and no cyanosis), Group C (PAH with a “coincidental” congenital shunt) and Group D (PAH following congenital defect repair) (1). This classification is essential and clinically useful, as it separates PAH-CHD groups by pathophysiology, clinical presentation, natural history and treatment options (1). All patients should, thus, be classified into one of these groups for clinical and research purposes, though refinements in the definitions and inclusion criteria are needed to ensure that all patients can be classified correctly and managed accordingly.

In this issue, Wacker et al. propose changes to the current classification of PAH-CHD, mainly focusing on two areas. They address the issue of PAH in patients with an atrial septal defect (ASD), which has long been a clinical conundrum. ASDs allow left-to-right shunting, thus causing an increase in pulmonary blood flow and, potentially, significant volume but not pressure overload to the pulmonary circulation (though pressure may increase as a result of the excessive pulmonary blood flow, a condition recently defined as “unclassified PH” by the 2024 PH World Symposium) (2). Shear stress to pulmonary arteries is far inferior to that posed by large post-tricuspid shunts; hence, only a small minority of patients with an ASD develop pulmonary vascular disease (PVD) characterised by a rise in PVR, in contrast to patients with a large post-tricuspid shunt in whom PVD is ubiquitous within a few years after birth (3, 4). The timing of diagnosis of PAH, and the type of RV (mal-)adaptation are also more in keeping with idiopathic PAH, which may suggest an independent mechanism responsible for the development of PAH in patients with an ASD, possibly “triggered” by the shunt (3, 4). These patients are currently included in group A when the PVD is severe enough to cause shunt reversal and hypoxia, or group B (1). Wacker et al. propose that these patients should always be classified under group C, where the CHD defect is considered a “bystander”. While a move from groups A or B to group C recognises the fact that haemodynamics alone do not explain why these patients develop PAH, it completely dismisses any contribution of the shunt to its pathogenesis. Available data would, in fact, point towards a multifactorial aetiology, with the potential contribution of genetics (5–7).

Wacker et al. also address the issue of “transient” shunts, typically ventricular septal defects (VSDs) in patients with complete (d-) transposition of the great arteries (TGA) who undergo timely repair (currently arterial switch procedure), but still develop PAH. The authors propose a new group in the PAH-CHD classification i.e., “group E”, which was also endorsed by the PH World symposium (8). Indeed, even large VSDs would not be expected to cause PVD when repaired early, though significant variability exists in terms of individual susceptibility to PVD and pulmonary haemodynamics in early life (9). Moreover, it is not clear why any (isolated) VSD, without TGA, could not fulfil criteria for inclusion in the new group E. Or indeed, why a patient with PAH after timely TGA (or VSD) repair cannot fall under group D, where patients are classified if PAH is diagnosed early or late after repair, regardless of the background CHD. This debate demonstrates the potential overlap between the established PAH-CHD groups, and the need for clarification and consensus.

**Figure 1:**
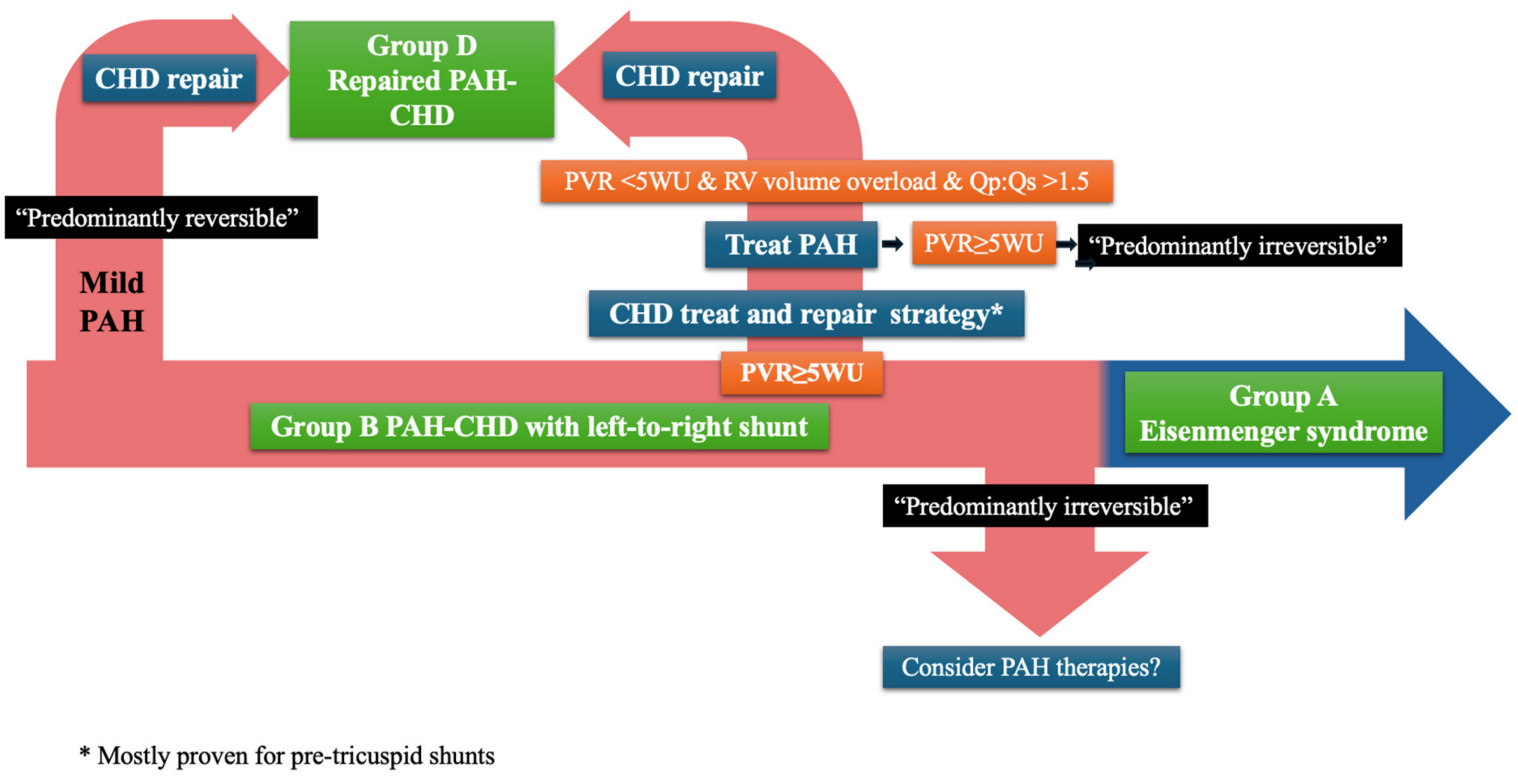
Classification and management of PAH-CHD. CHD, congenital heart disease; PAH, pulmonary arterial hypertension; PVR, pulmonary vascular resistance; WU, wood units; RV, right ventricle.

Ferrero et al. further enhance the need for clarification of the PAH-CHD classification, with a focus on group B. They provide a systematic description of the spectrum of phenotypes included in the group and propose further classification into “predominantly reversible” vs. “predominantly irreversible” PVD (Figure 1). The former includes patients previously classified as “correctable” but recognises the possibility of (at least partial) regression of PAH after repair (in patients with milder forms of PVD, as described by Heath and Edwards, and Wagenvoort) (10–12), but also the potential for residual PAH, while acknowledging the role of the “treat and repair” approach (that uses PAH therapies to “reverse” some of the PAH). “Predominantly irreversible” group B PAH-CHD identifies patients with more advanced PVD but not yet Eisenmenger syndrome (i.e., without hypoxia/cyanosis), in whom the defect should be left open and the focus should fall on managing PAH, though evidence of the use of PAH therapies is lacking.

Both Ferrero et al. and Wacker et al. highlight the difference in presentation and pathophysiology between patients with pre- vs. post-tricuspid shunts in group B, and overall PAH-CHD, and they list several established culprit genes, including mutations in the SOX-17 and TBX4 genes. Novel, rare mutations are continuously emerging, with one such example in the paper by Wang et al. in this issue of Frontiers: they describe a chromosome 2p16.1p15 microduplication mutation in an infant with an ASD who developed significant PAH. The microduplication has been reported in another 12 cases, but this was the only case in which PAH developed, while another two cases had an isolated ASDs without PAH. Ba et al. in this issue also describe PAH in a patient with an ASD and Kabuki syndrome. Once again, the ASD was felt to be a bystander and coincidental to the development of PAH-CHD, which was rapidly progressive.

These cases highlight the overlap between ASDs and PAH. Though there is a clear association, it cannot be explained by haemodynamics alone, and genetics may provide answers. Enhancing our understanding of the role of genetics in this setting, not only provides a potential explanation for the development of PAH, but will also allow better risk stratification and genetic counselling. PAH-CHD, and CHD in general, are likely to follow the path of cardiomyopathies, a group of conditions in which genetics has risen to a crucial role in clinical management (13).
